# Kindlin-2 interacts with β-catenin and YB-1 to enhance *EGFR* transcription during glioma progression

**DOI:** 10.18632/oncotarget.12439

**Published:** 2016-10-04

**Authors:** Yunwei Ou, Zitong Zhao, Weimin Zhang, Qingnan Wu, Chuanyue Wu, Xuefeng Liu, Ming Fu, Nan Ji, Dan Wang, Jiaji Qiu, Liwei Zhang, Chunjiang Yu, Yongmei Song, Qimin Zhan

**Affiliations:** ^1^ Department of Neurosurgery, Beijing Tiantan Hospital, Capital Medical University, Beijing 100050, China; ^2^ State Key Laboratory of Molecular Oncology, Cancer Institute and Cancer Hospital, Chinese Academy of Medical Sciences and Peking Union Medical College, Beijing 100021, China; ^3^ Beijing Neurosurgical Institute, Capital Medical University, Beijing 100050, China; ^4^ Department of Neurosurgery, Beijing Sanbo Brain Hospital, Capital Medical University, Beijing 100093, China; ^5^ Department of Pathology, University of Pittsburgh, Pittsburgh, PA 15261, USA; ^6^ Department of Biology and Shenzhen Key Laboratory of Cell Microenvironment, South University of Science and Technology of China, Shenzhen, 518055, China; ^7^ China National Clinical Research Center for Neurological Diseases, Beijing 100050, China

**Keywords:** EGFR, glioma, Kindlin-2, transcription

## Abstract

Kindlin-2 promotes carcinogenesis through regulation of cell-cell and cell-extracellular matrix adhesion. However, the role of Kindlin-2 in glioma has not been elucidated. We investigated Kindlin-2 expression in 188 human glioma tissue samples. High Kindlin-2 expression was correlated with high pathological grade and a worse prognosis. Kindlin-2 promoted glioma cell motility and proliferation both *in vitro* and *in vivo*. Importantly, Kindlin-2 activated the EGFR pathway and increased EGFR mRNA levels. In addition to up-regulating Y-box binding protein-1 (YB-1) and β-catenin expression, Kindlin-2 formed a transcriptional complex with YB-1 and β-catenin that bound to the EGFR promoter and enhanced transcription. The β-catenin/YB-1/EGFR pathway was required for Kindlin-2-mediated functions. Our data provide a better understanding of the mechanisms underlying glioma progression, and suggest that Kindlin-2 may be a biomarker and therapeutic target in glioma.

## INTRODUCTION

Glioma is the most common and deadliest type of primary brain tumor [1]. Recent advances in microsurgical therapy, radiotherapy, chemotherapy, and other glioma therapeutic strategies have improved the survival rate [2–3]. Prolonged exposure to ionizing radiation is a risk factor for glioma [4]. DNA copy number alterations, chromosomal rearrangements, methylation alterations, genetic variants, and oncogenic fusions have been identified in glioma [5–7]. However, the exact molecular mechanisms responsible for glioma growth, invasion, therapeutic resistance, and genomic instability have not been elucidated [8–9].

Kindlin-2 (mitogen inducible gene-2 [mig-2]) mediates cell-cell and cell-extracellular matrix adhesion. Kindlin-2 activates integrins and participates in several physiological processes such as chondrogenesis [10–11], cardiac muscle formation [12], myogenesis [13], and embryonic development [14]. Recently, Kindlin-2 was shown to be involved in tumor progression. Additionally, Kindlin-2 overexpression was proposed to be a prognostic biomarker for patients with hepatocellular carcinoma [15]. Guo et al. demonstrated that Kindlin-2 was highly expressed in breast cancer and found that Kindlin-2 interacted with and stabilized EGFR to regulate breast cancer progression [16–17]. Overexpression of Kindlin-2 was also observed in non-small cell lung [18], pancreatic [19], and gastric cancer [20]. However, Kindlin-2 overexpression decreased proliferation and migration in colorectal carcinoma cells, while Kindlin-2 down-regulation promoted tumorigenicity *in vitro* and *in vivo* [21]. These may be attributed to the expression and function of Kindlin-2 varying among different types of cancers. We previously reported that Kindlin-2 attenuated cisplatin-induced apoptosis in human glioma cells *in vitro* through the AKT/JNK and AKT/p38 signaling pathways [22]. However, whether Kindlin-2 has a critical role in glioma progression is unclear.

In this study, we analyzed Kindlin-2 protein expression in 188 glioma tissue samples, and found that Kindlin-2 expression was correlated with tumor grade and prognosis. We also demonstrated that Kindlin-2 promoted glioma cell growth and motility *in vitro* and *in vivo*. Finally, we determined that Kindlin-2 formed a transcriptional complex with Y-box binding protein-1 (YB-1) and β-catenin that enhanced *EGFR* transcription and promoted glioma cell proliferation, migration, and invasion.

## RESULTS

### High Kindlin-2 expression is correlated with high tumor grade and poor prognosis in glioma patients

**Figure 1:**
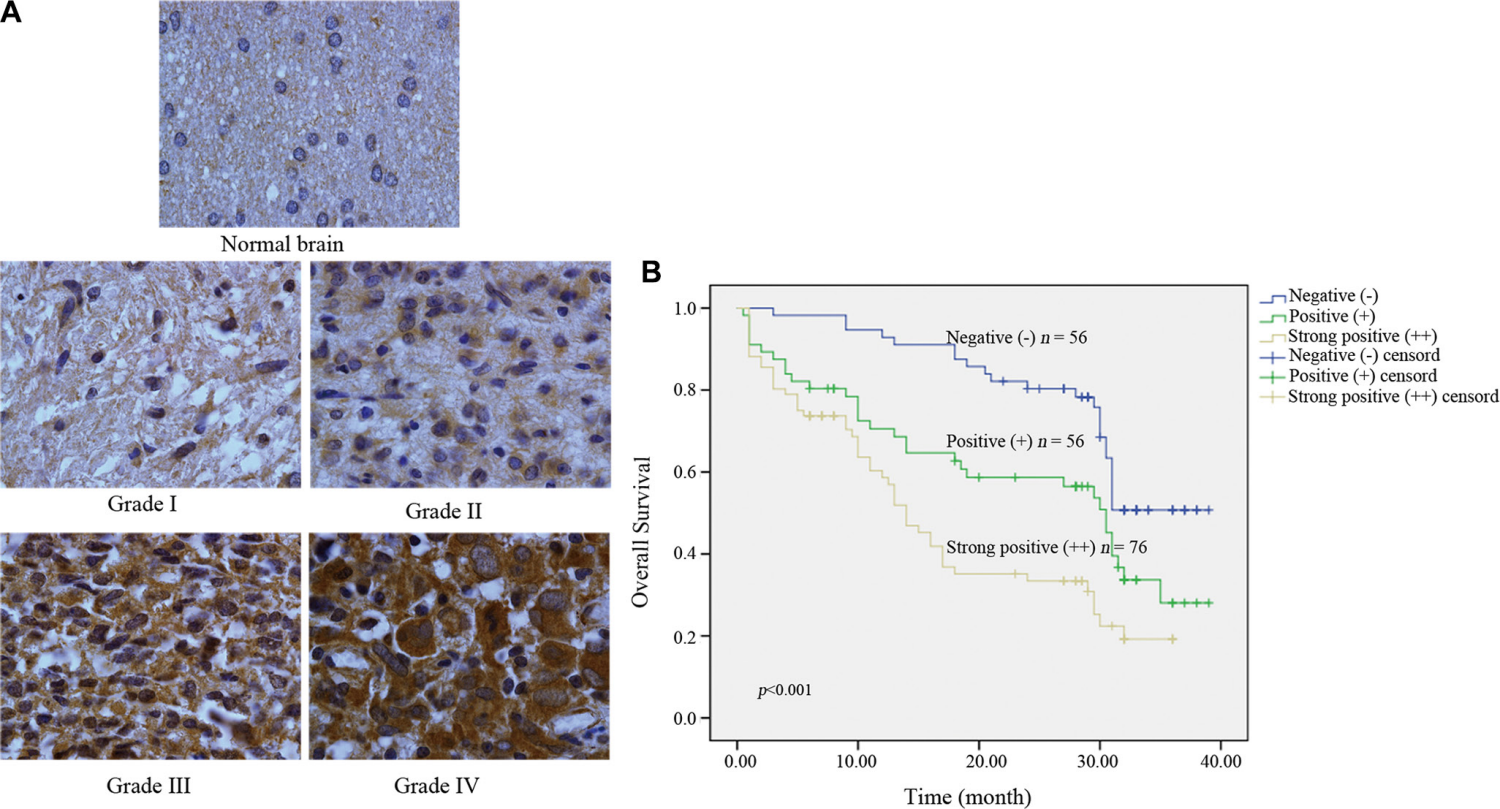
Kindlin-2 expression in intracranial glioma samples and the effects of Kindlin-2 expression on patient prognosis (**A**) Kindlin-2 expression in normal brain compared to glioma tissue (×400). (**B**) Kaplan-Meier curves with univariate analyses (log-rank) for patients with negative Kindlin-2 expression (blue line, *n* = 56), positive Kindlin-2 expression (green line, *n* = 56), and strong Kindlin-2 expression (brown line, *n* = 76).

We evaluated Kindlin-2 expression in 188 glioma and 10 normal brain tissue samples by immunohistochemistry. Stronger immunoreactivity was observed in glioma compared to normal brain tissue (Figure 1A). Among 188 tumor tissue samples, there were 76 (40.4%) with strong positive expression, 56 (29.8%) with positive expression, and 56 (29.8%) with negative expression. We then analyzed the correlation between clinicopathological parameters and Kindlin-2 expression in the 188 tissue samples. Kindlin-2 expression did not show a significant correlation with age or sex (*p* = 0.060 and *p* = 0.634, respectively). However, differences in Kindlin-2 expression were observed between high (III–IV) and low (I–II) pathological grades (*p* < 0.001) (Table 1). Higher Kindlin-2 expression was correlated with high pathological grade. Additionally, Kindlin-2 expression was associated with tumor location (*p* < 0.001) and clustered in the basal ganglia and thalamus (Table 2).

**Table 1:** The correlation between clinicopathological parameters and Kindlin-2 expression in 188 glioma cases

Clinicopathological parameters	Cases	Kindlin-2 expression^a^			*p*
		−	+	++^b^	
Age (y)					0.060
≤ Mean (39.0)	98	36	29	33	
> Mean	90	20	27	43	
Sex					0.634
Male	103	28	33	42	
Female	85	28	23	34	
Tumor grade					< 0.001
I–II	85	48	28	9	
III–IV	103	8	28	67	

a Data are shown as the number of cases;

b −, negative expression; +, positive expression; ++, strong positive expression.

**Table 2:** The correlation between tumor location and Kindlin-2 expression

Location	Cases	Kindlin-2^a^			*p*^c^	p′^d^				
		−	+	++^b^		Temporal lobe	Frontal lobe	Basal ganglia and thalamus	Parietal and occipital lobes	Brainstem
					< 0.001					
Temporal lobe	34	11	8	15			0.696	0.004	0.302	0.056
Frontal lobe	54	21	14	19				< 0.001	0.329	0.179
Basal ganglia and thalamus	45	2	14	29					0.003	< 0.001
Parietal and occipital lobes	29	8	12	9						0.122
Brainstem	26	14	8	4						
	188	56	56	76						

a Data are shown as the number of cases;

b −, negative expression; +, positive expression; ++, strong positive expression;

c *p* < 0.05 vs. control;

d *p'*< 0.005 vs. control.

We next investigated the relationship between Kindlin-2 expression and patient prognosis. In the Kindlin-2 (++) group, 64.5% (49/76) of the patients died from glioma. In the Kindlin-2 (+) group, 57.1% (32/56) died from glioma. Finally, in the Kindlin-2 (−) group, 41.1% (23/56) died of glioma (*p* = 0.027). Analysis of the median follow-up time indicated that Kindlin-2 (++) patients typically survived for 10.5 months, while Kindlin-2 (+) and Kindlin-2 (−) patients typically survived for 21.5 and 30 months, respectively (*p* < 0.001) (Table 3). Kaplan-Meier analysis demonstrated that age and sex did not significantly impact overall survival (*p* = 0.177 and *p* = 0.967, respectively) (Table 4). However, high Kindlin-2 expression was correlated with a worse prognosis (*p* < 0.001) (Figure 1B). Univariate and multivariate Cox proportional survival analyses were performed to analyze the possible interactive effects between clinicopathological variables and Kindlin-2 status on glioma patient prognosis. We determined that pathological grade and Kindlin-2 status were independent risk factors (hazard ratio [HR] = 1.764, 95% confidence interval [CI] = 1.076–2.892, *p* = 0.024; HR = 1.496, 95% CI = 1.109–2.0219, *p* = 0.008, respectively) (Table 5).

**Table 3:** The effect of Kindlin-2 status on outcome for the entire cohort of patients for whom follow-up data was available

Prognosis	Cases	Kindlin-2 expression^a^			*p*
		− (*n* = 56)	+ (*n* = 56)	++ (*n* = 76)	
Alive	84	33 (58.9)	24 (42.9)	27 (35.5)	
Dead	104	23 (41.1)	32 (57.1)	49 (64.5)	0.027
Median follow-up		30	21.5	10.5	< 0.001

a Data are shown as the number of cases (%).

**Table 4:** Kaplan-Meier estimates of the overall survival rate for 188 patients with glioma according to age, sex, Kindlin-2 status, and histological grade during the follow-up period of 0.5–39 months

Variable	Survival rate (%) ^a^	Log-rank test
Age (y)		0.177
≤ 39.0 (*n* = 98)	44 (44.9)	
> 39 (*n* = 90)	40 (44.4)	
Sex		0.967
Male (*n* = 103)	49 (47.6)	
Female (*n* = 85)	35 (41.2)	
Pathological grade		< 0.001
I–II (*n* = 85)	47 (55.3)	
III–IV (*n* = 103)	37 (35.9)	
Kindlin-2 status		< 0.001
− (*n* = 56)	33 (58.9)	
+ (*n* = 56)	24 (42.9)	
++ (*n* = 76)	27 (35.5)	

a Data are shown as the number of cases (%).

**Table 5:** Univariate and multivariate analyses using Cox proportional hazards models

Variable	Univariate analysis		Multivariate analysis	
	HR (95% CI)	*p*	HR (95% CI)	*p*
Age (≤ 39.0 *vs.* > 39.0)	1.298 (0.882–1.912)	0.186	–	–
Sex (male vs. female)	1.008 (0.686–1.482)	0.968	–	–
Tumor location	1.053 (0.936–1.185)	0.388	–	–
Pathological grade (I–II vs. III–IV)	2.598 (1.733–3.894)	< 0.001	1.764 (1.076–2.892)	0.024
Kindlin-2 status (negative vs. positive)	1.828 (1.430–2.336)	< 0.001	1.496 (1.109–2.019)	0.008

### Kindlin-2 promotes glioma cell migration, invasion, and proliferation *in vitro* and *in vivo*

**Figure 2:**
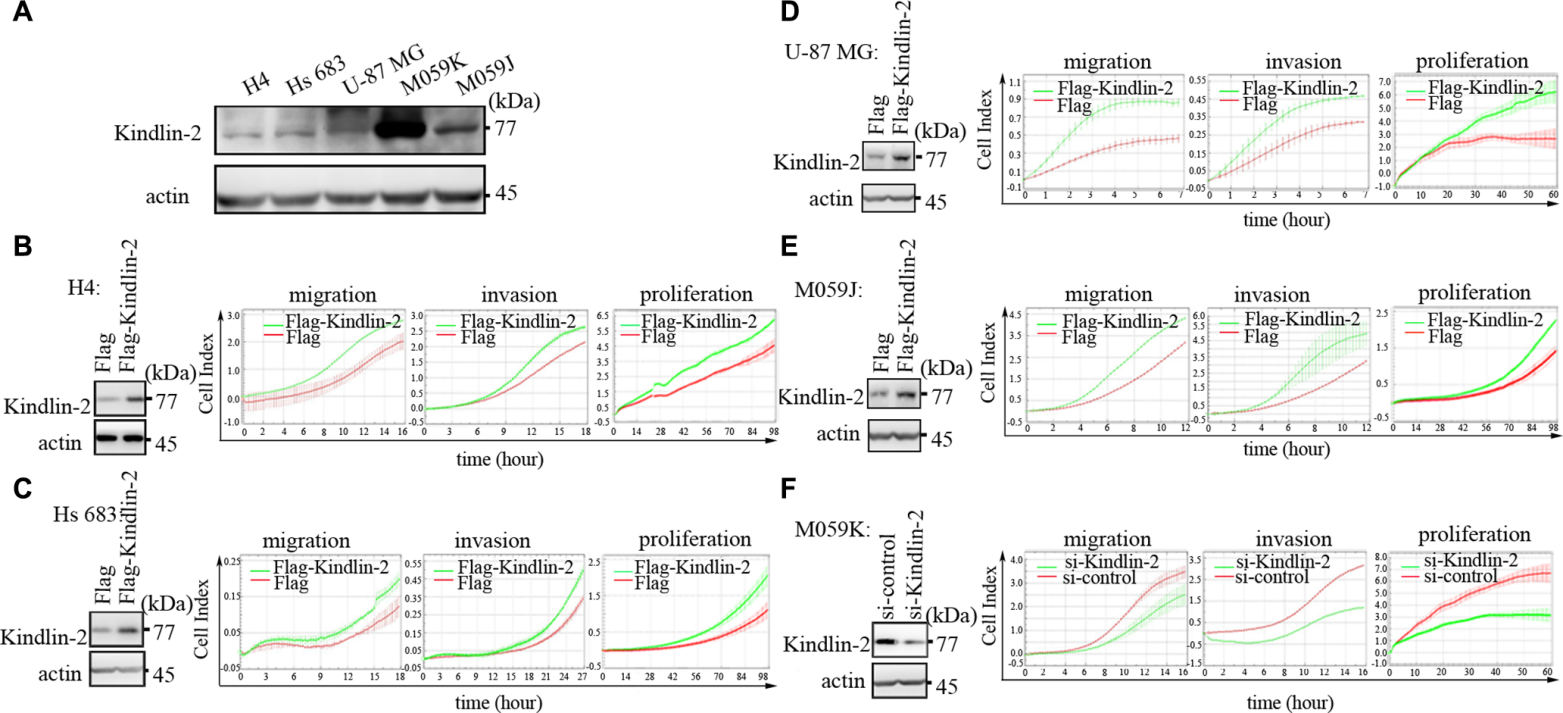
Kindlin-2 promotes glioma cell proliferation, migration, and invasion *in vitro* (**A**) Analysis of Kindlin-2 expression in H4, Hs 683, U-87 MG, M059K, and M059J glioma cell lines by western blotting. (**B**–**E**) Ectopic expression of Kindlin-2 in H4, Hs 683, M059J, and U-87 MG cells analyzed by western blotting (left). Overexpression of Kindlin-2 enhances glioma cell proliferation, migration, and invasion (right). (**F**) Western blot analysis of Kindlin-2 knockdown in M059K cells by siRNA (left). Kindlin-2 knockdown decreases glioma cell proliferation, migration, and invasion (right). Data are presented as the mean ± SD from three assays performed in triplicate.

We first investigated the expression of endogenous Kindlin-2 in H4, Hs 683, U-87 MG, M059K, and M059J glioma cells by western blotting. All glioma cell lines expressed Kindlin-2. However, M059K cells displayed the highest Kindlin-2 expression (Figure 2A). Therefore, the H4, Hs 683, U-87 MG, and M059J cell lines were optimal for experiments involving ectopic expression of Kindlin-2 through transient transfection of the cells with either Flag-tagged-Kindlin-2 (Flag-Kindlin-2) or an empty vector control. The M059K cells were suitable for experiments in which Kindlin-2 was depleted by siRNA.

We next examined the effects of Kindlin-2 on glioma cell migration and invasion using transwell assays. Kindlin-2 overexpression in H4, Hs 683, U-87 MG, and M059J cells increased migration and invasion compared to empty vector. Knockdown of endogenous Kindlin-2 by siRNA attenuated migration and invasion compared to control siRNA in M059K cells. Overexpression of Kindlin-2 in H4, Hs 683, U-87 MG, and M059J glioma cells enhanced the proliferative capacity. Conversely, siRNA-mediated knockdown of Kindlin-2 in M059K cells decreased the proliferative capacity (Figure 2B–2F).

**Figure 3:**
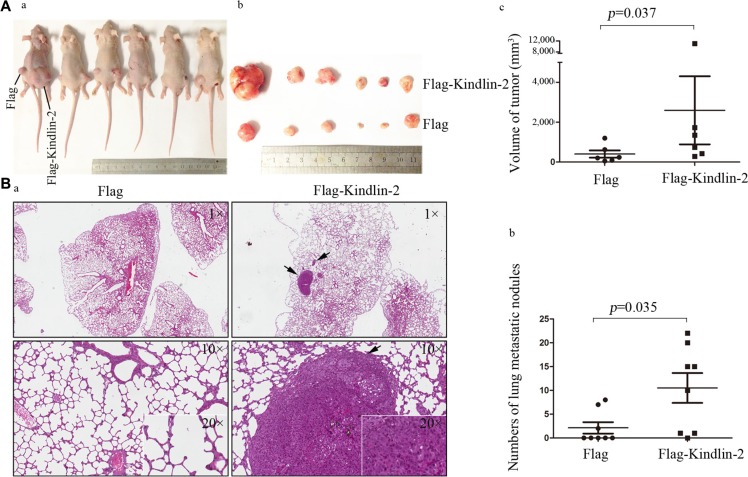
Kindlin-2 promotes glioma cell growth and metastasis *in vivo* (**A**) U-87 MG cells that stably expressed Kindlin-2 or control lentiviral vector were subcutaneously injected into athymic nude mice. The mice were sacrificed by day 30 and tumor formation assessed. (a). Mice were subcutaneously injected with Flag- (left side) or Flag-Kindlin-2-expressing cells (right side) and nursed for 30 days. (b). After 30 days, the tumors were resected and analyzed. c. Comparison of tumor volume between Kindlin-2 and control mice. The tumor volume was calculated as follows: (major circumference × minor circumference^2^)/2. (**B**) Overexpression of Kindlin-2 promotes glioma metastasis *in vivo*. a. Mice were sacrificed and the lungs harvested for H&E staining 8 weeks after tail vein injection with U-87 MG cells that stably expressed Kindlin-2 or control lentiviral vector. Representative images were acquired at 1×, 10× and 20× magnification (the arrows mark metastatic tumor nodules). (b) Quantification of microscopic nodules in the lungs of mice in each group. The error bars represent the SD. *p < 0.05 was considered significant.

To analyze the function of Kindlin-2 *in vivo*, we established U-87 MG cells that stably expressed Flag-Kindlin-2 or a control lentiviral vector. Mice were subcutaneously injected with either Flag- or Flag-Kindlin-2-expressing U-87 MG cells and nursed for 30 days. Forced expression of Kindlin-2 enhanced tumor formation and tumor volume compared to control cells (*p* = 0.037) (Figure 3A). We next analyzed the effects of Kindlin-2 on invasion using *in vivo* metastasis assays. U-87 MG cells that stably expressed Flag-Kindlin-2 or a control lentiviral vector were injected into mice via the tail vein. After 8 weeks, a higher number of microscopic tumor nodules were detected in the lungs of mice in the Flag-Kindlin-2 group compared to the Flag group (*p* = 0.035) (Figure 3B).

### Kindlin-2 activates EGFR signaling and binds to the *EGFR* promoter to enhance transcription

**Figure 4:**
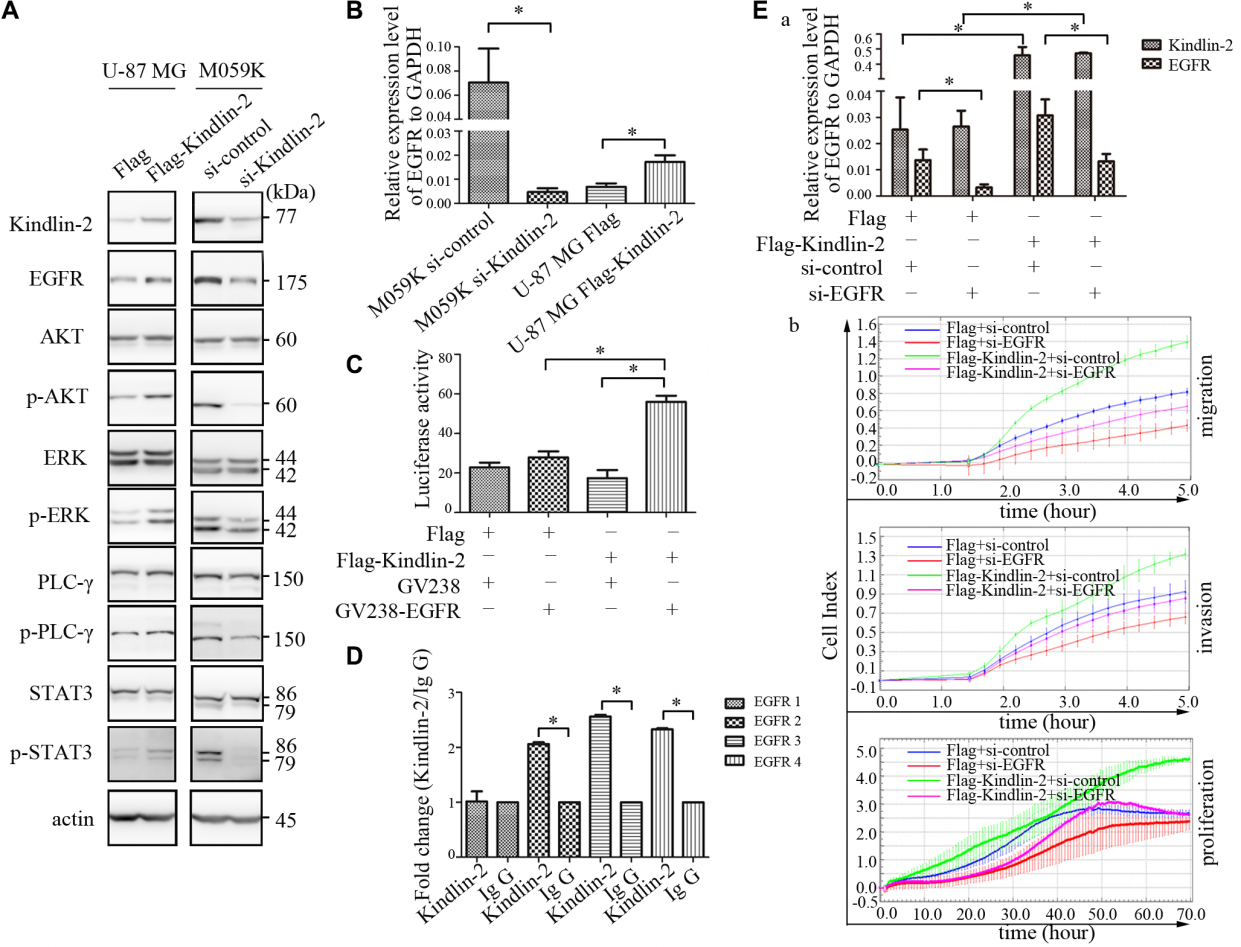
Kindlin-2 activates EGFR signaling and promotes EGFR transcription (**A**) Western blot analysis of endogenous EGFR, AKT, ERK, PLC-γ, and STAT3 levels in U-87 MG cells that overexpressed Kindlin-2 or control vector, and in M059K cells transfected with siRNA against Kindlin-2 or control siRNA. Protein expression was normalized to β-actin. (**B**) EGFR mRNA levels in M059K cells measured by real-time PCR after depletion of Kindlin-2 by siRNA, or ectopic expression of Kindlin-2 in U-87 MG cell lines. EGFR mRNA levels were quantified relative to GAPDH. (**C**) Dual luciferase reporter assays in U-87 MG cells were performed by co-transfecting a reporter plasmid containing the *EGFR* promoter and an expression plasmid encoding Flag-Kindlin-2 or Flag. *Photinus* luciferase activity was measured relative to *Renilla*. (**D**) ChIP assays to evaluate binding of Kindlin-2 to the *EGFR* promoter in U-87 MG cells. Four sites in the *EGFR* promoter (EGFR 1–4) were assessed. (**E**) Co-transfection of U-87 MG cells with anti-EGFR siRNA and an expression plasmid encoding either Flag-Kindlin-2 or Flag. EGFR and Kindlin-2 mRNA levels were analyzed by real-time PCR (a). Migration, invasion, and proliferation assays (b). Data are shown as the mean ± SD from three assays performed in triplicate. *p < 0.05 was considered significant.

EGFR is frequently activated in glioma [23]. Additionally, Kindlin-2 can stabilize EGFR in breast cancer [17]. Therefore, we hypothesized that Kindlin-2 could regulate EGFR signaling in glioma. Immunohistochemical analysis revealed higher EGFR expression in subcutaneous xenografts of mice in the Flag-Kindlin-2 compared to the control group (*p* = 0.001, *r* = 0.816, Supplementary Figure S1). We next transiently transfected Flag-Kindlin-2 or control plasmids into U-87 MG cells. In parallel, siRNA targeting Kindlin-2 was transfected into M059K cells. The transfection efficiency was confirmed by western blotting. Overexpression of Kindlin-2 enhanced EGFR expression in U-87 MG cells. Conversely, knockdown of Kindlin-2 by siRNA reduced EGFR protein levels in M059K cells (Figure 4A). To determine whether Kindlin-2 activated signaling downstream of EGFR, we evaluated the activation state of four EGFR-mediated signaling pathways. Up-regulation of Kindlin-2 promoted AKT, ERK, PLC-γ, and STAT3 phosphorylation but did not alter the total protein levels. In contrast, Kindlin-2 knockdown by siRNA decreased AKT, ERK, PLC-γ, and STAT3 phosphorylation compared to controls, but had no effect on total protein expression (Figure 4A).

Both transcriptional activation and post-transcriptional modifications can increase EGFR expression. [24] Therefore, we analyzed EGFR mRNA levels by real-time PCR. Overexpression of Kindlin-2 in U-87 MG cells resulted in an increase in the levels of EGFR mRNA. In contrast, depletion of Kindlin-2 by siRNA in M059K cells reduced EGFR mRNA levels (Figure 4B). These data indicated that Kindlin-2 regulated *EGFR* transcription in glioma cells. To confirm these findings, a reporter plasmid containing the first 2 kb of the *EGFR* promoter was constructed for luciferase reporter assays. Overexpression of Kindlin-2 increased EGFR luciferase activity compared to control U-87 MG cells (Figure 4C).

To determine whether Kindlin-2 could bind to the *EGFR* promoter, we designed four primers within the first 2 kb of the start site of EGFR for chromatin immunoprecipitation (ChIP) assays using U-87 MG cells. The first primer was positioned downstream of the *EGFR* start site while primers 2–4 were upstream [25]. The Kindlin-2 antibody effectively immunoprecipitated DNA amplified by EGFR primers 2–4 compared to the immunoglobulin (IgG) controls, whereas the real-time PCR product amplified by the EGFR 1 primer was not significantly detected compared to the IgG control (Figure 4D). These results indicated that Kindlin-2 bound to the upstream region of the *EGFR* promoter and regulated transcription.

We transiently transfected U-87 MG cells with the Flag-Kindlin-2 plasmid along with an anti-EGFR siRNA to determine whether the Kindlin-2-induced effects were dependent on the level of EGFR mRNA. The level of EGFR mRNA was significantly reduced in EGFR knockdown cells even in the presence of the siRNA (Figure 4E, a). Kindlin-2-mediated glioma cell proliferation, migration, and invasion were abolished upon EGFR depletion by siRNA (Figure 4E, b). These results suggested that *EGFR* transcriptional activity was required for Kindlin-2 function.

### Kindlin-2 regulates *EGFR* transcription through interaction with YB-1 and β-catenin

YB-1 is a transcription factor that binds to the *EGFR* promoter in breast cancer cells [26–27]. Kindlin-2 can form a tripartite transcriptional complex with β-catenin and T-cell factor 4 (TCF4) to enhance Wnt signaling through expression of Wnt target genes such as Axin2, Cyclin D1, LEF1, Twist, and MMP2 [28]. Therefore, we hypothesized that Kindlin-2 could regulated *EGFR* transcription in glioma cells through its interaction with β-catenin and YB-1.

**Figure 5:**
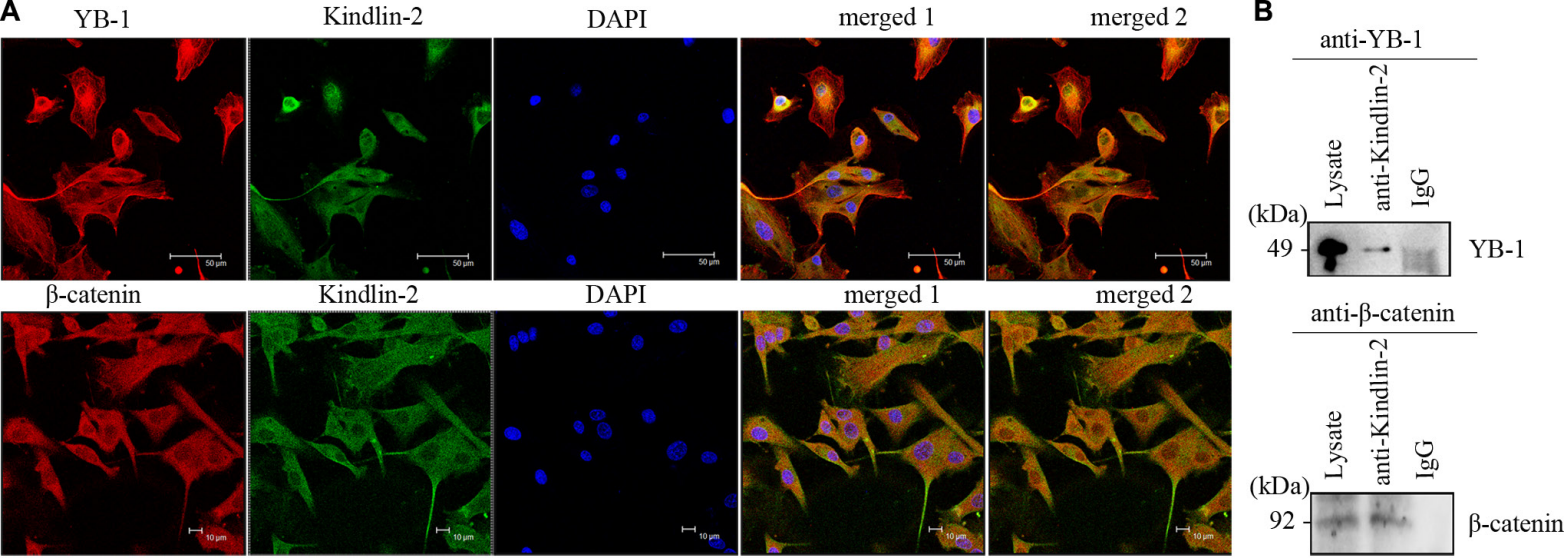
Kindlin-2 forms a complex with YB-1 and β-catenin (**A**) Confocal microscopy indicates Kindlin-2, YB-1, and β-catenin co-localize in U-87 MG cells. Cells were double stained with an anti-Kindlin-2 monoclonal antibody (green) and an anti-YB-1 polyclonal antibody (red), or an anti-β-catenin polyclonal antibody (red). DNA was stained with DAPI (blue). Kindlin-2, YB-1 (or β-catenin), and DAPI images were merged in merged image 1. Kindlin-2 and YB-1 (or β-catenin) were merged in merged image 2. Yellow dots in merged 1 and merged 2 reflect Kindlin-2 and YB-1 (or β-catenin) co-localization. Scale bar: 50 μm (top) or 10 μm (bottom). (**B**) Immunoprecipitation of Kindlin-2 with YB-1 and β-catenin. Lysates of human U-87 MG cells were mixed with a mouse anti-Kindlin-2 antibody. The immunoprecipitates were analyzed by western blotting with anti-YB-1 (top) or β-catenin (bottom) antibodies.

Using confocal microscopy, we determined that endogenous Kindlin-2, YB-1, and β-catenin predominantly co-localized in the cytoplasm of U-87 MG cells, although nuclear localization was also observed (Figure 5A). We next immunoprecipitated Kindlin-2 from U-87 MG cell lysates using an anti-Kindlin-2 antibody. Both YB-1 and β-catenin were detected in the Kindlin-2-immunoprecipitated complexes (Figure 5B). Thus, endogenous Kindlin-2 formed a complex with YB-1 and β-catenin.

### YB-1 and β-catenin are required for Kindlin-2-mediated *EGFR* transcription

**Figure 6:**
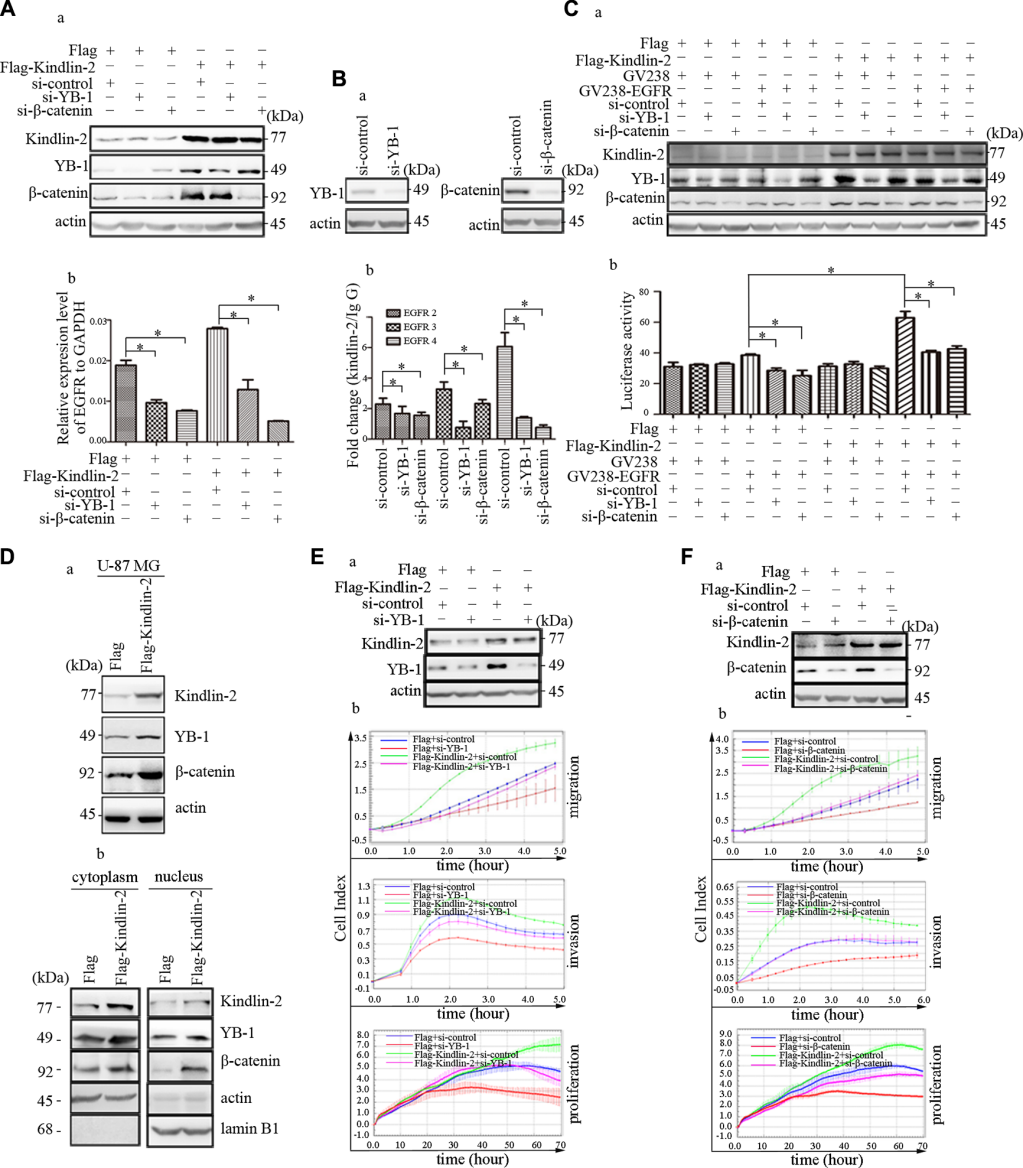
YB-1 and β-catenin are required for Kindlin-2-mediated *EGFR* transcription and function in glioma (**A**) Analysis of EGFR mRNA levels by real-time PCR after co-transfection of U-87 MG cells with anti-YB-1 siRNA (or anti-β-catenin siRNA) and an expression plasmid encoding Flag-Kindlin-2 or Flag. Cells were harvested for western blotting to assess the transfection efficiency (a). EGFR mRNA expression was quantified relative to GAPDH (b). (**B**) YB-1 or β-catenin were knocked down by siRNA in U-87 MG cells. The cells were then harvested for western blotting to analyzed the transfection efficiency (a). ChIP assays were performed to examine the effects of YB-1 and β-catenin in the binding regions of Kindlin-2 to the promoter of *EGFR* 2–4 (b). (**C**) The reporter plasmid containing the *EGFR* promoter was co-transfected with an expression plasmid encoding Flag-Kindlin-2 and either anti-YB-1 or anti-β-catenin siRNA) into U-87 MG cells. The cells were harvested for western blotting to calculate assess the transfection efficiency (a). Dual luciferase reporter assays were then performed. *Photinus* luciferase activity was measured relative to *Renilla* (b). (**D**) Kindlin-2 positively regulates the expression of YB-1 and β-catenin and promotes their nuclear location. Western blot analysis of Kindlin-2, YB-1, and β-catenin expression in U-87 MG cells that overexpressed Kindlin-2 or control vector. Protein expression levels were normalized to β-actin (a). The distributions of Kindlin-2, YB-1, and β-catenin in the cytoplasm and nucleus were analyzed using a Nuclear and Cytoplasmic Extraction kit after transfection of U-87 NG cells with an expression plasmid encoding either Flag-Kindlin-2 or Flag. Cytoplasmic protein expression was normalized to β-actin, while nuclear protein expression was normalized to Lamin B1 (b). (**E**–**F**) Co-transfection of U-87 MG cells with anti-YB-1 or -β-catenin siRNA and an expression plasmid encoding either Flag-Kindlin-2 or Flag. Western blots showing the transfection efficiency (a). Analysis of glioma cell migration, invasion, and proliferation (b). Data are presented as the mean ± SD from three assays performed in triplicate. *p < 0.05 was considered significant.

We investigated whether Kindlin-2-mediated *EGFR* transcription was dependent on its association with YB-1 and β-catenin. U-87 MG cells were transiently transfected with Flag-Kindlin-2 and either anti-YB-1 or-β-catenin siRNA. We confirmed that down-regulation of YB-1 or β-catenin did not affect Kindlin-2 expression (Figure 6A, a). Knockdown of YB-1 or β-catenin reduced EGFR mRNA levels (Figure 6A). Additionally, depletion of endogenous YB-1 or β-catenin in U-87 MG cells by siRNA (Figure 6B, a) resulted in a decrease in amplification of the immunoprecipitated DNA by EGFR primers 2–4 compared to the IgG control group in ChIP assays (Figure 6B, b). We co-transfected the EGFR reporter, Flag-Kindlin-2, and anti-YB-1 or -β-catenin siRNA into U-87 MG cells and found that knockdown of either YB-1 or β-catenin reduced Kindlin-2-induced EGFR luciferase activity (Figure 6C). These data indicated that YB-1 and β-catenin were necessary for Kindlin-2 regulation of *EGFR* transcription.

Increased nuclear localization of active factors can promote *EGFR* transcription. Although Kindlin-2 localized to the nucleus, whether Kindlin-2 controlled the nuclear localization of YB-1 and β-catenin was unclear. U-87 MG cells were transiently transfected with either Flag-Kindlin-2 or empty vector. Ectopic expression of Kindlin-2 in U87-MG cells enhanced YB-1 and β-catenin expression compared to controls (Figure 6D, a). We next analyzed the expression of Kindlin-2, YB-1, and β-catenin in nuclear and cytoplasmic extracts by western blotting. Although all three proteins were predominantly detected in the cytoplasm, the data indicated they could also localize to the nucleus. Overexpression of Kindlin-2 increased the levels of nuclear YB-1 and β-catenin (Figure 6D, b).

To investigate the functional association between Kindlin-2, YB-1, and β-catenin, U-87 MG cells were co-transfected with either anti-YB-1 or -β-catenin siRNA and Flag-Kindlin-2. Knockdown of YB-1 or β-catenin individually abolished Kindlin-2-induced glioma cell proliferation, migration, and invasion (Figure 6E–6F). These data were consistent with the results of EGFR knockdown in Kindlin-2-overexpressing cells (Figure 4E). Collectively, the results indicated that Kindlin-2 formed a complex with YB-1 and β-catenin to regulate *EGFR* transcription and promote glioma progression.

## DISCUSSION

In this study, we demonstrated that Kindlin-2 expression was positively correlated with clinical tumor grade in human glioma tissue samples. Kindlin-2 status was an independent risk factor, suggesting that Kindlin-2 expression can be an indicator of glioma patient prognosis. We also determined that Kindlin-2 promoted glioma cell motility and proliferation *in vitro*, and that it could promote growth and invasion *in vivo*. Finally, Kindlin-2 formed a tripartite transcriptional complex with YB-1 and β-catenin, which bound to the *EGFR* promoter and enhanced *EGFR* transcription and glioma cell proliferation and motility.

Kindlin-2 functions in several signaling pathways to promote tumorigenesis in breast [16–17], non-small cell lung [18], and pancreatic cancer [19]. Opposite functions have been described in mesenchymal tumors, serous epithelial ovarian cancer, and colorectal cancer [21, 29–30]. We found that Kindlin-2 expression was higher in glioma compared to normal brain tissue, and that high Kindlin-2 expression was correlated with high pathological grade. These data indicated that increased Kindlin-2 expression could promote glioma cell proliferation and/or motility. Kaplan-Meier analysis revealed that positive Kindlin-2 expression was associated with poor survival, and that Kindlin-2 was an independent prognostic factor in glioma. Overall, the data suggest that Kindlin-2 expression could be a useful biomarker in glioma.

Although previous studies have indicated that age is a prognostic factor in glioma [31–32], we did not observe an association between age and survival, and kindlin-2 expression in our cohort. This may be explained by the relatively young age of the patients (average age, 39 years). The number of patients should be increased and the age span/distribution noted in future studies. Associations between tumor location and tumor-specific biomarkers have been described previously. For example, high vascular endothelial growth factor (VEGF) was associated with glioblastoma located in the left frontal lobe and right caudate [33–34]. We determined that glioma tissue samples from the basal ganglia and thalamus had higher Kindlin-2 expression than other regions of the brain. This data suggests that gliomas that arise in the basal ganglia and thalamus may have enhanced growth and motility capacity, which could result in a poor prognosis.

Abnormal cell growth and motility are hallmarks of glioma and other malignant tumors [35]. Interestingly, Kindlin-2 has been shown to promote cell adhesion, motility, and proliferation in several cancers [20–21, 36–37]. We found that ectopic expression of Kindlin-2 promoted glioma cell growth and invasion *in vivo*. Our data has demonstrated that Kindlin-2 promotes glioma progression through inducing glioma cell motility and proliferation. Kindlin-2 participates in tumorigenesis through multiple mechanisms including EGFR signaling. The major signaling pathways downstream of EGFR include the MEK-ERK, PI3K-AKT, PLC-PKC, and STAT pathways [38]. EGF can stimulate Kindlin-2 expression at both the mRNA and protein levels through the EGFR signaling pathway [17, 39]. The Kindlin-2/integrin β1/AKT axis was shown to contribute to esophageal squamous cell cancer, while the Kindlin-2/EGFR/AKT axis was shown to be involved in breast cancer [39–40]. In renal tubular epithelial cells (TECs), Kindlin-2 can recruit Sos-1 to regulate Ras activation by activating ERK1/2 and AKT signaling, which promotes TEC epithelial-mesenchymal transition (EMT) [41]. The EGFR signaling pathway also plays a critical role in glioma. *EGFR* amplification has been observed in 30–50% of glioblastomas, which is the highest grade glioma [42–43]. These data support the hypothesis that Kindlin-2 is involved in glioma development through regulation of EGFR signaling. Indeed, we found that forced expression of Kindlin-2 in glioma cells increased EGFR expression, while Kindlin-2 depletion decreased EGFR levels in glioma cells.

Several mechanisms are responsible for dysregulation of EGFR expression including genomic alterations and both transcriptional and posttranscriptional modifications [24]. Guo et al. showed that Kindlin-2 altered EGFR protein levels, but not mRNA levels, in breast cancer cells [17]. However, we determined that ectopic expression of Kindlin-2 increased EGFR mRNA levels, which was significantly decreased in Kindlin-2 -depleted glioma cells, suggesting that Kindlin-2 regulated *EGFR* transcription. Kindlin-2 is an important transcriptional regulator of multiple genes including *Axin2*, *Cyclin D1*, *LEF1*, *Twist*, *MMP2*, *sFRP1,* and *Sox9* [11, 28]. Using ChIP and luciferase reporter assays, we demonstrated that Kindlin-2 bound to the upstream region of the *EGFR* promoter and positively regulated *EGFR* transcription. The Kindlin-2-induced effects were attenuated by EGFR knockdown, suggesting that *EGFR* transcription is required for Kindlin-2 function in glioma.

Although the clinical importance of EGFR and many of the proteins that regulate *EGFR* transcription is clear (e.g. c-Jun, SP-1), the underlying mechanisms have not been fully elucidated [44–45]. Recently, YB-1, an oncogenic transcription/translation factor, was shown to regulate *EGFR* transcription in breast cancer [25, 27]. YB-1 is overexpressed in many cancers such as breast cancer, lung cancer, and glioma [46–48]. Kindlin-2, which regulates Wnt signaling, also regulates β-catenin expression, and forms tripartite transcriptional complex with β-catenin and TCF4 to promote Wnt target gene expression during breast cancer progression [28]. We hypothesized that the Kindlin-2/β-catenin/YB-1 complex could regulate *EGFR* transcription in glioma. Using confocal microscopy and immunoprecipitation, we demonstrated an interaction between Kindlin-2, YB-1, and β-catenin in glioma cells. Simultaneous up-regulation of Kindlin-2 and silencing of YB-1 or β-catenin in glioma cells resulted in a decrease in EGFR mRNA levels and transcriptional activity, and disruption of the Kindlin-2 binding sites in the *EGFR* promoter. Thus, Kindlin-2 forms a tripartite transcriptional complex with YB-1 and β-catenin to promote *EGFR* transcription.

We determined that Kindlin-2 enhances YB-1 and β-catenin protein expression. However, downregulation of YB-1 or β-catenin did not alter Kindlin-2 expression, suggesting that Kindlin-2 functions upstream of both β-catenin and YB-1. However, the increase in *EGFR* transcriptional activity was associated with enhanced nuclear localization of trans-activating elements. Kindlin-2, YB-1, and β-catenin all exhibited nuclear localization in glioma cells, which confirmed that up-regulation of Kindlin-2 enhanced the nuclear localization of YB-1 and β-catenin. These results indicated that Kindlin-2 induces YB-1 and β-catenin expression and nuclear localization to promote *EGFR* transcription. Kindlin-2 was also shown to promote *FoxM1* expression, which could also induce the nuclear localization of β-catenin in glioma cells [28, 49]. These data suggest that Kindlin-2 may promote YB-1 and β-catenin nuclear translocation through FoxM1. Finally, we determined that YB-1 and β-catenin were required for Kindlin-2-mediated migration, invasion, and proliferation in glioma cells. Therefore, Kindlin-2/YB-1/β-catenin/EGFR signaling is critical for glioma development.

In summary, our results indicate that Kindlin-2 is up-regulated in glioma cells and acts as an oncogene. It is an independent risk factor for poor prognosis. The Kindlin-2/YB-1/β-catenin complex promotes *EGFR* transcription and contributes to glioma progression. Kindlin-2 is a potential diagnostic and prognostic marker in glioma, and inhibition of Kindlin-2 may be a novel strategy for glioma treatment.

## MATERIALS AND METHODS

### Patient tissue

A total of 188 glioma specimens were collected from 103 male and 85 female glioma patients (average age of 39.0 years) who underwent treatment for glioma at the Sanbo Brain Hospital at Capital Medical University between 2008 and 2010. Ten normal brains tissue specimens (mostly medulla) were donated by individuals who died in traffic accidents. Patient selection methods and follow-up criteria were described previously [50]. Informed consent was obtained from the patients and the study was approved by the Institutional Research Ethics Committee.

### Immunohistochemistry

Tissue sections were deparaffinized in xylene and rehydrated in graded ethanol. The sections were first incubated with a 1:100 dilution of anti-Kindlin-2 (or anti-EGFR) of primary antibody at 4°C overnight, and then incubated with a horse radish peroxidase (HRP)-conjugated secondary antibody for 1 h. Sections were stained with 3,3′-diaminobenzidine. The samples were scored as previously described [50].

### Subcutaneous and experimental metastasis animal models

BALB/c nude mice (female, 5 weeks old) were purchased from Beijing Vital River Laboratories (Beijing, China). We subcutaneously injected BALB/c nude mice (on the back sides) with 2 × 10^6^ U-87 MG cells that stably expressed Kindlin-2 or the control lentiviral vector (GeneChem, Shanghai, China) and analyzed tumor growth. The mice were sacrificed after 30 d and tumor volume was estimated: V = (major circumference × minor circumference^2^)/2. Cells were injected into mice via the tail vein for metastasis assays. The mice were sacrificed after 8 weeks, and the lungs were enucleated and paraffin embedded. Sections were collected and stained with hematoxylin and eosin (H&E). Animal experiments were performed according to the Institutional Animal Care and Use Committee guidelines of the Experimental Animal Center of the Cancer Institute and Cancer Hospital of the Chinese Academy of Medical Sciences and Peking Union Medical College.

### Cell culture

Human glioma cell lines (U-87 MG, H4, Hs 683, M059K, and M059J) were purchased from the Cell Culture Center (Chinese Academy of Medical Sciences, Beijing, China). The M059K and M059J cell lines were purchased from the ATCC (Washington, DC, USA). All cell lines were validated by short tandem repeat assays before use. U-87 MG cells were grown in MEM supplemented with 10% fetal bovine serum (FBS). The H4, Hs 683, M059K, and M059J cells were cultured in DMEM supplemented with 10% FBS. All cells were cultured at 37°C in a 5% CO_2_ environment.

### Antibodies

The anti-EGFR, -Akt, -pAkt, -STAT3, -pSTAT3, -Erk1/2, -pErk1/2, -PLC-γ1, -pPLC-γ1, -YB-1, -β-catenin, -Lamin B1, and -β-actin antibodies were purchased from Cell Signaling Technology, Inc. (Danvers, MA, USA). The anti-Migfilin antibody was a gift from Dr. Cary Wu (University of Pittsburgh, Pittsburgh, PA, USA). The HRP-conjugated anti-rabbit and anti-mouse antibodies, Rhodamine (TRITC)-conjugated Affinipure goat anti-rabbit IgG antibody, Alexa Fluor 488-conjugated Affinipure goat anti-mouse IgG antibody, and 4′,6′-diamidino-2-phenylindole (DAPI) were purchased from Sigma (St. Louis, MO, USA).

### Plasmid and siRNA transfection

The Kindlin-2 plasmid was a gift from Dr. Cary Wu. The siRNAs targeting Kindlin-2, EGFR, YB-1, and β-catenin were custom designed and purchased from Ribobio (Guangzhou, China). Cells were transfected with either the plasmids or siRNAs using Lipofectamine 2000 (Invitrogen, Carlsbad, CA, USA) using the manufacturer's protocols.

### Real-time PCR

Real-time PCR was performed using the Premix Ex Taq kit (Takara) and a 7300 Real-Time PCR System (Life Technologies) according to the manufacturer's instructions. The primers were the following: used were as follows:

Kindlin-2: Forward, 5′-CGAGAATCTTGGAGGCCCAT -3′,

Reverse, 5′-TATTTGGGGGTAGGGGGAGG-3′;

EGFR: Forward, 5′-GGTGACCGTTTGGGAGTTGA-3′,

Reverse, 5′-CCCTGAATGACAAGGTAGCG-3′;

GAPDH: Forward, 5′-TGTTGCCATCAATGACCCCTT-3′,

Reverse, 5′-CTCCACGACGTACTCAGCG-3′.

### Western blotting and immunoprecipitation

Western blotting and immunoprecipitation analyses were performed as described [50]. For immunoprecipitation, lysates were incubated with the primary antibody followed by protein A-agarose beads (Invitrogen). The immune complexes were washed and resuspended in sample buffer for western blotting.

### Proliferation, migration, and invasion assays

Cancer cell proliferation, migration, and invasion were monitored with a xCELLigence Real-Time Cell Analyzer (RTCA)-MP/SP (Acea Biosciences/Roche Applied Science) according to the manufacturer's instructions. We resuspended 2,000–3,000 cells in 100 μL of culture medium for proliferation assays and 4–10 × 10^4^ cells in 100 μL of serum-free medium for migration and invasion assays.

### Luciferase reporter assays

U-87 MG cells were co-transfected with a reporter plasmid (GV238 vector) containing the *EGFR* promoter (GeneChem, Shanghai, China) and an expression plasmid encoding Flag-Kindlin-2 using Lipofectamine 2000 (Invitrogen). We co-transfected the cells with pRL-CMV *Renilla* (Promega) to standardize the transfection efficiency. Luciferase activity was measured using the Dual-Luciferase reporter assay (Promega) and the manufacturer's protocol. *Photinus* luciferase activity was measured relative to *Renilla*.

### ChIP assays

ChIP assays were peformed using a ChIP assay kit (Roche, Switzerland) according to the manufacturer's instructions. The *EGFR* promoter, which spanned regions within the first 2 kb of the start site, was amplified using the following primers [25].

EGFR-1: Forward, 5′-TCGCCGCCAACGCCA CAAC-3′,

Reverse, 5′-ACACGCCCTTACCTTTCT TTTCCTCCAG-3′;

EGFR-2: Forward, 5′-CCGCGAGTTTCCC TCGCATTTCT-3′,

Reverse, 5′-CCTTCCCCCTTTCCCTTCTTTTGTTTTAC-3′;

EGFR-3: Forward, 5′-TCCCATTTGCCTTTCTCTAGTTT TGTTTTC-3′,

Reverse, 5′-GTCCACCCCATCCCCACTGTTCCTTCTC-3′;

EGFR-4: Forward, 5′-TTCAGCAAACCCATTCTTCT-3′,

Reverse, 5′-GCTTCCTGCACACCTGGGCTGAG-3′

### Confocal microscopy

Cells were grown on coverslips for 24 h and then fixed in cold methanol for 15 min. The cells were incubated with the primary antibodies (diluted 1:100) overnight at 4°C, and then stained with the second antibodies and DAPI. Immunofluorescence was analyzed using a confocal laser scanning microscope (Leica, Germany).

### Statistical analysis

Statistical analyses were performed with the SPSS 11.5 software. Correlations between the degree of staining and the subgroups according to clinicopathological classifications were calculated using the Pearson's χ^2^ test. The Kaplan-Meier method was used to estimate the overall survival rate as a function of time. Survival differences were analyzed using log-rank tests. Cox proportional hazards models were used for univariate and multivariate analyses of prognostic factors. Data are shown as the mean ± standard deviation (SD) and were analyzed using two-sided student's *t* tests. *P* values < 0.05 were considered significant.

## SUPPLEMENTARY MATERIALS FIGURES


