# Supplementary material for: Kindlin-2 interacts with β-catenin and YB-1 to enhance EGFR transcription during glioma progression
Source: Oncotarget. 2016 Oct 4;7(46):74872–85. doi: 10.18632/oncotarget.12439 (PMC5342708; doi:10.18632/oncotarget.12439)
Supplement: Supplementary file 1 [file oncotarget-07-74872-s001.pdf]

Kindlin-2 interacts with β-catenin and YB-1 to enhance *EGFR* transcription during glioma progression

Supplementary Materials

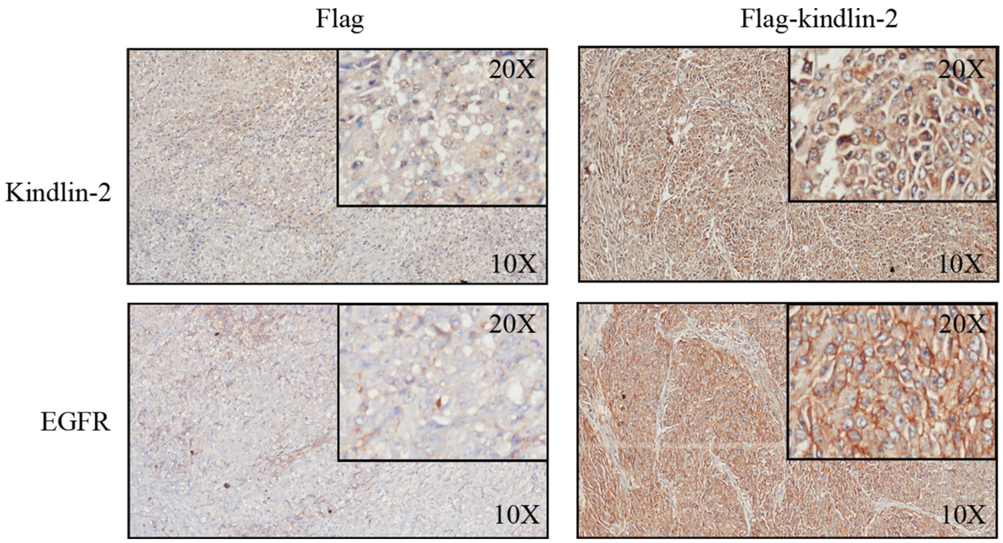

Supplementary Figure S1: The expression of Kindlin-2 and EGFR in the subcutaneous xenografts of mice. The expression of Kindlin-2 and EGFR were determined by immunohistochemistry, and images were taken on a microscope at 10× and 20× magnification.
